# Structure of the bacteriophage PhiKZ non-virion RNA polymerase

**DOI:** 10.1093/nar/gkab539

**Published:** 2021-06-28

**Authors:** Natàlia de Martín Garrido, Mariia Orekhova, Yuen Ting Emilie Lai Wan Loong, Anna Litvinova, Kailash Ramlaul, Tatyana Artamonova, Alexei S Melnikov, Pavel Serdobintsev, Christopher H S Aylett, Maria Yakunina

**Affiliations:** Section for Structural and Synthetic Biology, Department of Infectious Disease, Imperial College London, London, UK; Peter the Great St. Petersburg Polytechnic University, St. Petersburg, Russia; Section for Structural and Synthetic Biology, Department of Infectious Disease, Imperial College London, London, UK; Peter the Great St. Petersburg Polytechnic University, St. Petersburg, Russia; Section for Structural and Synthetic Biology, Department of Infectious Disease, Imperial College London, London, UK; Peter the Great St. Petersburg Polytechnic University, St. Petersburg, Russia; Peter the Great St. Petersburg Polytechnic University, St. Petersburg, Russia; St. Petersburg State University, St. Petersburg, Russia; Section for Structural and Synthetic Biology, Department of Infectious Disease, Imperial College London, London, UK; Peter the Great St. Petersburg Polytechnic University, St. Petersburg, Russia; Sechenov Institute of Evolutionary Physiology and Biochemistry Russian Academy of Sciences, St. Petersburg, Russia

## Abstract

Bacteriophage ΦKZ (PhiKZ) is the archetype of a family of massive bacterial viruses. It is considered to have therapeutic potential as its host, *Pseudomonas aeruginosa*, is an opportunistic, intrinsically antibiotic resistant, pathogen that kills tens of thousands worldwide each year. ΦKZ is an incredibly interesting virus, expressing many systems that the host already possesses. On infection, it forms a ‘nucleus’, erecting a barrier around its genome to exclude host endonucleases and CRISPR-Cas systems. ΦKZ infection is independent of the host transcriptional apparatus. It expresses two different multi-subunit RNA polymerases (RNAPs): the virion RNAP (vRNAP) is injected with the viral DNA during infection to transcribe early genes, including those encoding the non-virion RNAP (nvRNAP), which transcribes all further genes. ΦKZ nvRNAP is formed by four polypeptides thought to represent homologues of the eubacterial β/β′ subunits, and a fifth with unclear homology, but essential for transcription. We have resolved the structure of ΦKZ nvRNAP to better than 3.0 Å, shedding light on its assembly, homology, and the biological role of the fifth subunit: it is an embedded, integral member of the complex, the position, structural homology and biochemical role of which imply that it has evolved from an ancestral homologue to σ-factor.

## INTRODUCTION

Bacteriophage ΦKZ is the prototypical member of a family of giant bacterial viruses (1,2). It infects *Pseudomonas aeruginosa*, which is an intrinsically antibiotic resistant opportunistic pathogen that kills tens of thousands annually. ΦKZ is therefore considered a potential candidate for medical use as a bacteriophage therapy (3,4). ΦKZ is of exceptional size with a massive DNA genome (280 334 bp) (5) encoding many proteins that the host would typically be expected to provide during infection. It also expresses several other systems that the host lacks entirely. During infection, ΦKZ forms a ‘nucleus’ within the host cytoplasm that protects its genome from host endonucleases, and which it traffics with a tubulin cytoskeleton (6,7). This implies intriguing evolutionary parallels to their eukaryotic equivalents, and suggests a possible viral origin for the eukaryotic nucleus and cytoskeleton.

The lifecycle of ΦKZ in *P. aeruginosa* is unaffected by the host RNA polymerase (RNAP) inhibitor rifampicin. This indicates that ΦKZ is independent of the host transcription machinery; ΦKZ provides its own RNA polymerases throughout the infection cycle (8). Whereas almost all known bacteriophage-encoded RNA polymerases are single subunits, ΦKZ is one of only a few viruses (9–11) to encode multisubunit RNAPs (msRNAPs). All known msRNAPs comprise the conserved double-ψ β-barrel (DPBB) domain in the two largest subunits, β and β’ in bacteria (12–15). These largest subunits are usually catalytically inactive without auxiliary subunits. In eubacteria, a dimer of α subunits and a single ω subunit are required to assemble the minimal catalytically active core capable of the RNA polymerisation reaction (ββ’α_2_ω) (16–19). Although this core is catalytically active, exchangeable σ-subunits are required to initiate transcription with specificity from their cognate promoters at the correct transcription initiation sites (13,20).

Bacteriophage ΦKZ encodes two sets of proteins homologous to small subsections of the two largest subunits of bacterial RNAP, β and β’. It is not believed to possess homologues of the α or ω subunits usually required for transcription at all. The ΦKZ virion RNAP is formed from at least four polypeptides: gp178, gp149, gp180 and gp80. This is injected with the genome to express ΦKZ early genes (8). The ΦKZ non-virion RNAP (nvRNAP) is formed from these early genes by five polypeptides: gp55, gp68, gp71–73, gp74 and gp123 (10). Both gp55 and gp74 exhibit a degree of homology to the β’ subunit, whereas gp71–73 and gp123 represent similarly weakly homologous counterparts of the β subunit. No homology had previously been identified for ΦKZ-gp68 (10). *In vitro* transcription assays have revealed that complexes lacking gp68 are unable to elongate existing RNA or bind to a DNA–RNA hybrid, demonstrating that gp68 is essential for even the most basic biological functions of the complex (21).

In this study, we have determined the three-dimensional structure of ΦKZ nvRNAP to better than 3 Å using cryogenic electron microscopy (cryo-EM), allowing atomic modelling of the majority of the complex. Our structure confirmed the previously predicted homology of the split β/β’-like subunits, but further revealed that subunit gp68 is integral to the ΦKZ nvRNAP complex, interacting with both gp71–73 and gp55. Biochemical studies of nvRNAP assembly implied that gp68 plays a decisive role in the formation of the active centre. We used DNA binding assays to demonstrate that gp68 is essential for both promoter binding and recognition, and structural comparison with other RNAPs showed that it has weak homology to, and is located in the same place as, bacterial σ factors.

## MATERIALS AND METHODS

### Cloning

The set of pET-based plasmids containing one of the phiKZ nvRNAP subunit genes, the pnvCo-Ex plasmid and the pGp68 plasmid, were generated previously (21). PACYC184 was digested using SalI, and blunt ends generated using Klenow fragment (Thermo Fisher Scientific, Waltham, MA, USA). The set of pACYC-based plasmids was created by blunt end insertion of PCR-products with sequences corresponding to the T7 promoter, lac operator, ribosome binding site, and requisite genes of the phiKZ nvRNAP subunit without an additional His-tag from the pET-set of plasmids. pET19K-Hisgp55-(T7pr_gp71-73), or pET28a-Hisgp68-(T7pr_gp71-73) plasmids were assembled from two PCR-fragments using the NEBuilder HiFi DNA Assembly Master Mix (New England Biolabs, Ipswich MA, USA) according to the manufacturer's protocol.

### Protein expression and purification

BL21(DE3) *Escherichia coli* cells were transformed with either one plasmid, or two plasmids with compatible origins. Expression was induced by the addition of 1 mM IPTG once an OD_600_ of 0.5–0.7 had been reached, and cultures were incubated at 22°C for 3 h after induction. Recombinant ΦKZ nvRNAP complexes were purified as previously described (21). For subunit complex validation, 1 g wet mass of each corresponding cell pellet was disrupted by sonication in 10 mL of buffer A (40 mM Tris–Cl pH 8.0, 10% glycerol, 500 mM NaCl,1 mM DTT) containing 5 mM Imidazole followed by centrifugation at 11 000 rcf for 30 min at 4°C. Clarified lysate was loaded onto a HisTrap HP 1 mL (GE LS, Chicago IL, USA) that had previously been equilibrated, and then washed with buffer A with 5 mM Imidazole. The recombinant complexes were then eluted with buffer A containing 250 mM Imidazole. Size-exclusion chromatography of the recovered factions was performed using a Superdex 200 Increase 10/300 GL (GE LS, Chicago, IL, USA) in TGED buffer (20 mM Tris–Cl pH 8.0, 5% glycerol, 0.5 mM EDTA, 1 mM DTT) with 200 mM NaCl.

For purification of Gp68 without a tag, 1 g wet mass of cell pellet was resuspended in 10 ml TGED buffer with 50 mM NaCl and disrupted by sonication. Clarified lysate was passed through a HiTrap Q XL column (GE LS, Chicago, IL, USA) equilibrated into the same buffer. The resulting sample was loaded onto HiTrap Heparin HP (GE LS, Chicago, IL, USA) followed by washing using TGED buffer with 100 mM NaCl. The protein was eluted using TGED buffer with 200 mM NaCl. The eluted fractions were concentrated (Amicon Ultra-4 Centrifugal Filter Unit with Ultracel-30 membrane, EMD Millipore, Merck, Burlington MA, USA) and purified by size exclusion chromatography using a Superdex 200 Increase 10/300 (GE LS, Chicago, IL, USA) in TGED buffer with 200 mM NaCl.

### DNA-template preparation

The RNA–DNA hybrid and all types of DNA templates were prepared as previously described (21). The oligonucleotides used are listed in Supplementary Table S4.

### Native PAGE and *in vitro* transcription

Native polyacrylamide gel electrophoresis and *in vitro* transcription reactions were performed as previously described (21).

### Electrophoretic mobility shift assay (EMSA) and photo-crosslinking

Electrophoretic mobility shift assay (EMSA) was carried out as previously described (21). For photo-crosslinking reactions the reaction mixture (10 μl) containing 5 pmol of the template DNA and 7.5 pmol of ΦKZ nvRNAP were incubated in 1x transcription buffer (40 mM Tris–Cl pH 8.0, 10 mM MgCl_2_, 5 mM DTT) for 15 min at 37°C. To induce the formation of covalent cross-links, the mixture was irradiated for 15 s. A RAPOP-100 femtosecond laser system and an ATsG800-17 third harmonic generator (‘Avesta-Project’) were used as a source of ultraviolet radiation with a wavelength of 266 nm. The radiation parameters (the 3rd harmonic) were: radiation pulse energy of 10 μJ, pulse duration of 80 fs, repetition frequency of 2 kHz, and beam diameter of 2 mm. The resulting average power was 20 mW, and the radiation power density in the pulse was 4 GW cm^–2^. To break down unsuccessfully crosslinked complexes, heparin was added to the reaction mixture to a concentration of 100 g/l and reaction mixture was incubated at 37°C for 10 min. Samples were analyzed by EMSA in DNA loading buffer (8.3% glycerol) or SDS-DNA loading buffer (8.3% glycerol, 0.2% SDS, 30 mM EDTA) before loading onto the 8% TBE–polyacrylamide gel.

### Limited proteolysis

ΦKZ nvRNAP (10 pmol), and its complexes with DNA-templates (20 pmol), were digested using trypsin (Sigma-Aldrich, St. Louis, MO, USA) in 100 mM Tris-Cl pH 8.5 at 22 °C for 30 min. The concentration of trypsin used was 0.09 mg/ml. The proteolysis reactions were quenched through the addition of SDS-PAGE sample buffer, followed by immediate incubation of each sample at 95°C for 5 min. The resulting samples were analyzed by SDS-PAGE (8 and 10% polyacrylamide gels).

### Mass-spectrometry

Protein bands of interest were manually excised from the Coomassie-stained SDS or native TBE–PAGE. Individual slices from SDS or native PAGE were prepared for mass-spectrometry through *in situ* trypsin digestion at 37°C for 4h as previously described (10). In the case of TBE-PAGE slice gels, no washing step was performed.

### Grid preparation

ΦKZ nvRNAP complexes were exchanged into sample buffer containing 15 mM Tris–Cl pH 8.0, 150 mM NaCl, 0.5 mM EDTA, 2 mM MgCl_2_, 1 mM DTT, to a final concentration of 0.3 mg/ml. Samples of ΦKZ nvRNAP were adsorbed to a thin film of graphene oxide deposited upon the surface of holey carbon copper grids (R2/1, 300 mesh, Quantifoil). Grids were blotted for 1–2 s before plunge freezing in liquid ethane using a Vitrobot Mark IV (Thermo Fisher Scientific, Waltham, MA, USA) at 4°C and 100% humidity.

### Calculation of reference density

A total of 257 micrographs from a cryo-grid of ΦKZ nvRNAP were collected on an early Falcon direct electron detector using an FEI Tecnai F20 electron microscope (Thermo Fisher Scientific, Waltham, MA, USA) at a magnification of 81 000-fold, an acceleration voltage of 200 kV, and a total dose of 50 e^–^/Å^2^ over a defocus range of –2 to –5 μm. A dataset of 45 980 particles was selected semi-automatically using BOXER (22). The parameters of the contrast transfer function were determined using CTFFIND4 (23). Particles were 2D-classified into 100 classes in two dimensions using RELION 3.0 (24) and seven well-defined classes including 18 508 particles were selected for initial 3D reconstruction. Initial models were generated using the stochastic gradient descent approach in RELION 3.0, then filtered to 50 Å and used as an initial reference for automatic refinement. Projections from the resulting initial models were consistent with class-averages, and were therefore used for further refinement of higher resolution data.

### Dataset acquisition

Data from ΦKZ nvRNAP samples was collected on a Titan Krios G3i (Thermo Fisher Scientific, Waltham, MA, USA) at the London Consortium for Electron Microscopy microscope sited at the Crick institute, equipped with a K3 direct electron detector (GATAN, San Diego, USA) and operated at 300 kV, 48 000-fold magnification and with an applied defocus range of –0.75 to –3.25 μm. Frames were recorded automatically using EPU, resulting in 10 741 images of with a pixel size of 0.85 Å on the object scale. Images were recorded as stacks of 32 separate frames in electron counting mode, comprising a total exposure of ∼51.2 e^–^ Å^–2^.

### Data processing

Frames were aligned, summed and weighted by dose according to the method of Grant and Grigorieff using MotionCor2 to obtain a final image. Poor-quality micrographs were rejected based on diminished estimated maximum resolution on CTF estimation using CTFFIND4 and visually based on irregularity of the observed Thon rings. Particles were selected using BATCHBOXER (25), and refinement thereafter performed using RELION 3.0.

Two-dimensional reference-free alignment was performed on 3 179 799 initial particles to exclude those that did not yield high-resolution class averages. Of these, 1 043 026 particles were retained for further refinement, and after a further screening based on CTF parameters for higher resolution, 855 016 contributed to an initial refinement to high resolution using the auto-refinement procedure in RELION 3.0, reaching just under 3 Å according to an independent half-set FSC of 0.143. These were then classified without alignment into nine classes in three-dimensions, the class with the largest ordered fraction of the molecule being retained for final high-resolution refinement. This final gold-standard refinement of ΦKZ nvRNAP from 50 085 particles reaching 3.3 Å according to an independent half-set FSC of 0.143.

### Modelling and refinement

Initial models for the most homologous regions of the ΦKZ nvRNAP were prepared using Swiss-Model based on PDB-6EDT, and FUGUE. The model was then rebuilt with COOT and extended outwards from these well-defined reference points to the remaining regions of the four homologous proteins. ΦKZ-gp68 was finally built into the remaining density, initially as a poly-alanine model, before the sequence register was eventually established based upon the identification of clear patterns of large side-chains and secondary structure elements (26). The atomic model was refined with PHENIX real-space refine. Homology searches and Cα comparisons were carried out using the DALI-lite server, while surface area calculations were performed using the PISA server.

## RESULTS AND DISCUSSION

**Figure 1.**
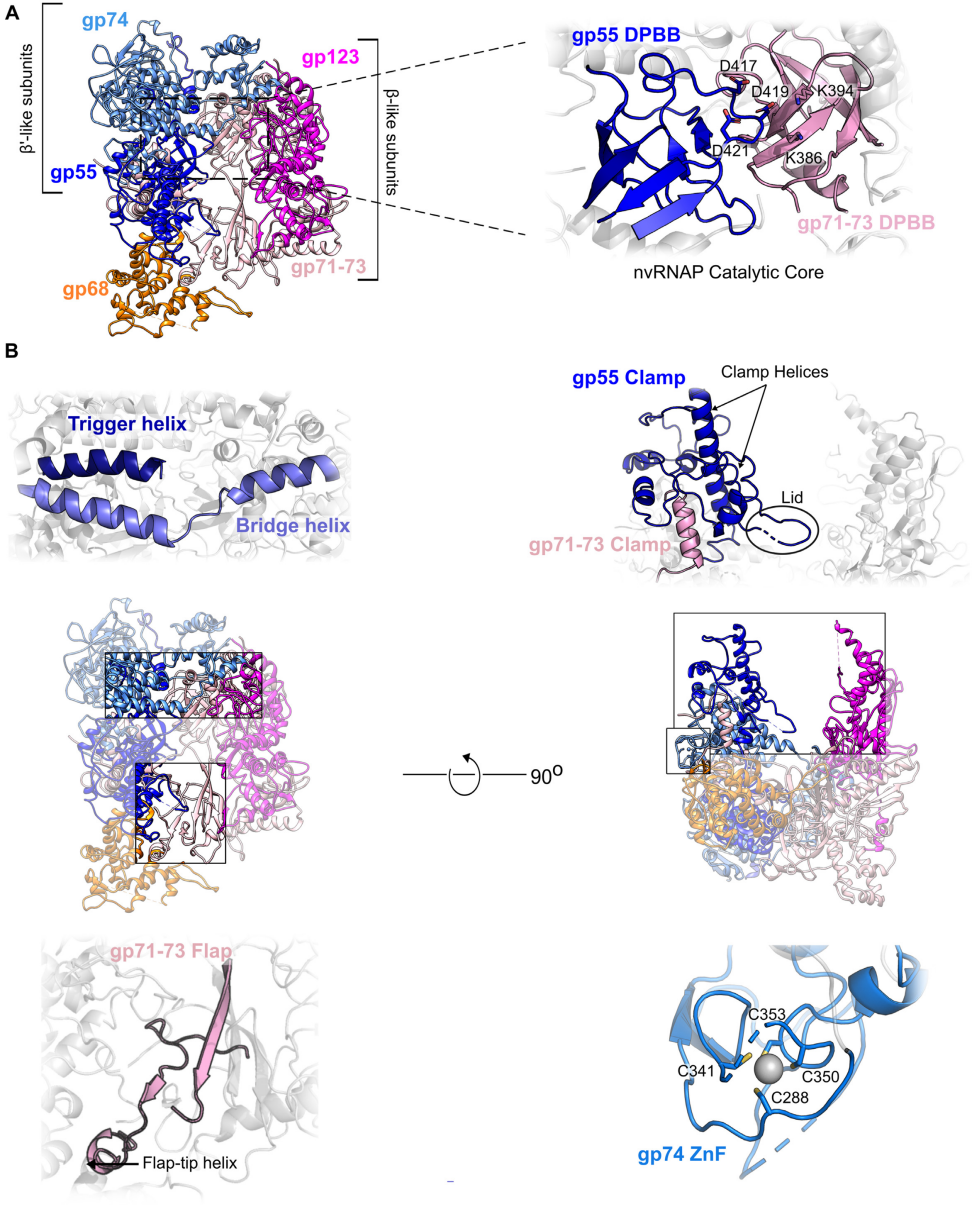
Overview and architectural elements of ΦKZ nvRNAP. (**A**) Molecular model of the ΦKZ nvRNAP in cartoon representation. (**B**) Insets: expanded views of: (top-right) DPBBs of gp55 (blue) and gp71–73 (pink); (centre-left) Trigger helix (deep blue) and bridge helix (light blue) of gp74; (centre-right) nvRNAP clamp (gp71–73 light pink; gp55 blue); (bottom-left) Flap gp71–73 (pink); (bottom-right) Zn-finger of gp74 (blue).

We generated recombinant ΦKZ nvRNAP complexes in *E. coli* using a co-expression system with one plasmid bearing the four β/β’-like proteins and another encoding gp68. This allowed the simple generation of both four- and five-polypeptide ΦKZ nvRNAP complexes, as well as free gp68, for biochemical experiments (Supplementary Figure S1). For structure determination, ΦKZ nvRNAP complexes were stabilised by adsorption to a graphene oxide film covering a holey carbon copper grid and vitrified before visualisation by cryo-EM. Electron micrographs of vitrified ΦKZ nvRNAP particles proved similar in appearance to eubacterial msRNAP (27). We resolved structures of the better ordered core regions of the complex by single particle analysis to a final resolution of just below 3.0 Å (FSC = 0.143), and a maximum of 73% of the complex to 3.3 Å (FSC = 0.143) after extensive sorting for the clamp (Supplementary Figures S2 and S3). Initial comparison of our structure with several previously resolved msRNAPs, in particular the closest homologue identified in the PDB, *Mycobacterium tuberculosis* RNAP (PDB: 6EDT) (28), allowed us to establish positions for the most conserved core regions of the β/β’-like subunits. We expanded upon these conserved segments, and then interpreted the remainder of the density to provide an atomic model covering the majority of the complex (Figure 1, Supplementary Figure S4, Supplementary Table S1).

The overall architecture of ΦKZ nvRNAP proved to resemble that found in eubacterial msRNAPs as expected given the previously reported sequence homology (Supplementary Figure S4). The ΦKZ nvRNAP complex retains the conserved β/β’ architecture characteristic of msRNAPs, with a shallow active site channel running along the elongated side of the structure (Figure 1). One side of the enzyme is formed by the β’-like subunits, gp55 and gp74, with the β-like subunits gp71-73 and gp123 forming the opposing side, the internal DNA channel running between them through the centre of the complex as expected, and illustrated by complementary electrostatics (Supplementary Figure S5). Density corresponding to the most peripheral parts of the enzyme which would encircle the channel is weakly resolved in our structure due to flexibility, presumably given the absence of nucleic-acid. Through examination of locally filtered densities and comparison to the eubacterial RNAP structure, we can assign the flexible regions that we were unable to model confidently to gp68, gp74 and gp123 (Supplementary Figure S6). One region of particular note is the unresolved region of gp123. The very proximal regions of this domain have a visually similar topology to eubacterial β Si1 (29), however no further residues can be assigned with confidence. We note that the presence of this domain would be consistent with an evolutionary origin in the proteobacteria, which is expected given the host spectra of ΦKZ and related bacteriophages.

The key active elements and enzymatic residues of ΦKZ nvRNAP are well-conserved. This implies an identical reaction cycle to eubacteria, despite the split and missing subunits. The DPBBs that form the catalytic core are contributed by gp55, which contains the universally conserved DxDGD motif (17,30) that coordinates the catalytic Mg^2+^ (^417^DFDGD^421^) between β5 (403I-M405) and β6 (Q422-L427), and by gp71–73, which contains the two conserved lysines (K386 and K394) believed to be responsible for the interaction with DNA (12) (Figure 1A). The structural elements that govern the msRNAP reaction cycle are also conserved in ΦKZ nvRNAP; β’-like subunit gp74 contains the ‘bridge’ helix (L242-Y279) and the ‘trigger’ helix/loop (Figure 1B), which switch between alternate conformations during nucleotide addition (30,31), although only the first helix of the ‘trigger’ loop (residues T365-V383) is well-ordered in our structure, as well as the conserved Zn^2+^ binding domain (C288, C341, C350, C353), known to interact with the promoter during transcription initiation (Figure 1B) (30). The ‘clamp’, a flexible element whose position is key to regulate the entry of single-stranded DNA into the active site, is also conserved and positioned similarly to its situation within the *M. tuberculosis* open promoter complex (RPo) structure (Figure 1B) (10,32). This region, lying within the gp55 N-terminal domain, exhibited particular flexibility, and was only ordered in the small fraction of the data yielding the most complete structure. We also observe a region corresponding to the eubacterial msRNAP ‘flap’ within gp71–73, however this area is very poorly ordered (Figure 1B).

**Figure 2.**
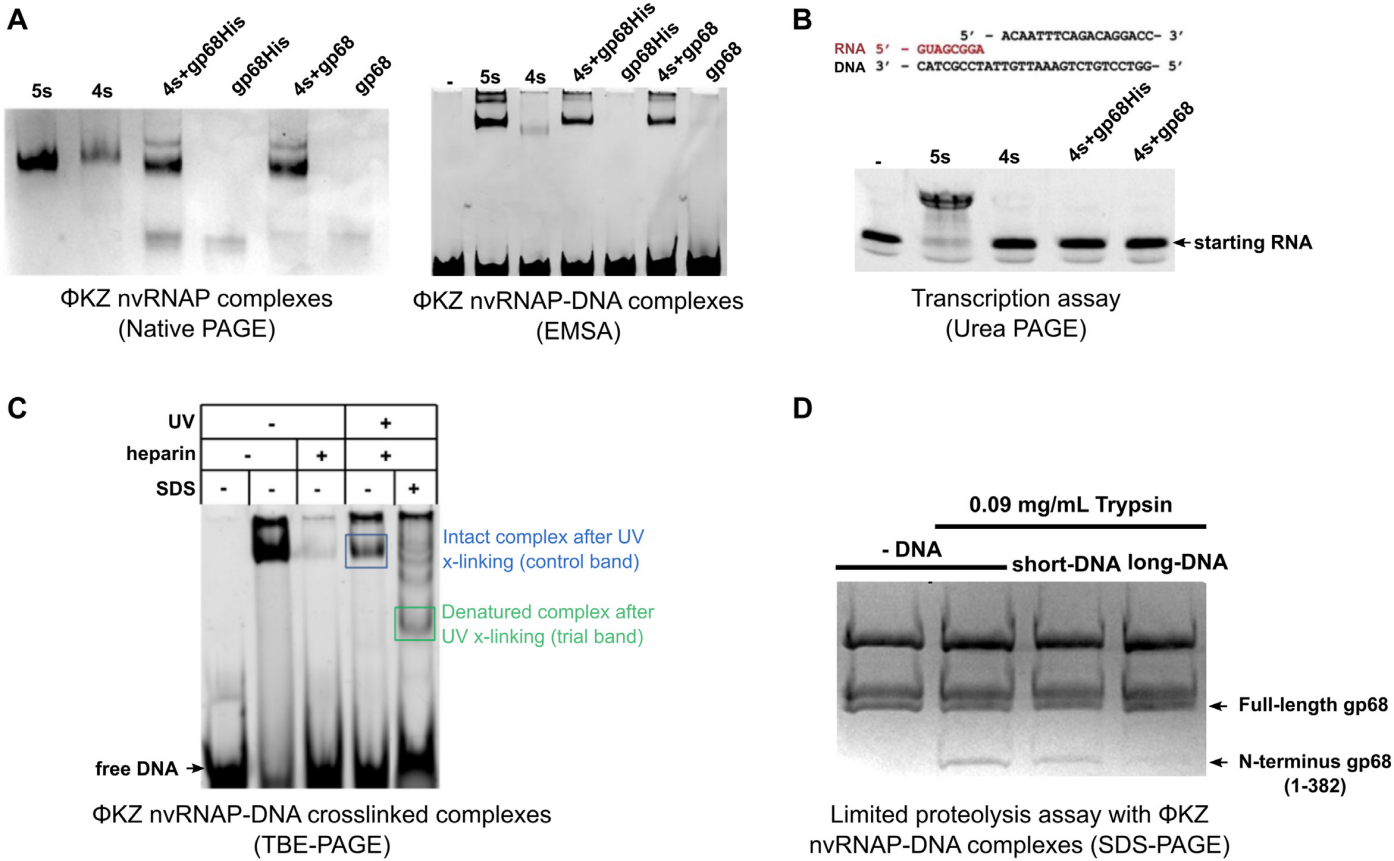
Gp68 is necessary for assembly of active polymerase, and binding of cognate DNA. (**A**) Native PAGE of 4, and 5 subunit complexes prepared through the inclusion of gp68 at different points, and EMSA with a 212 bp specific dsDNA-template containing the KZP119L promoter sequence for different constructs and assemblies of recombinant nvRNAP complexes and independently purified gp68-His and gp68 samples. (**B**) PAGE of the transcription assay from the RNA-DNA hybrid shown above. Activity is indicated by decreased mobility (lane 5s). (**C**) SDS-PAGE of UV cross-linked DNA bound complex, showing bands extracted for subsequent analysis by mass-spectrometry. (**D**) Protection of gp68 from limited proteolysis by DNA templates. Both short and long templates are based on the KZP119L promoter sequence; the short template extends from -12 to +15 nucleotides from transcription start marked +1, whereas the long template indicates a full 212 bp PCR-fragment. The full gp68 and the N-terminal fragment of gp68 extending to 382 residues are marked by arrows.

**Figure 3.**
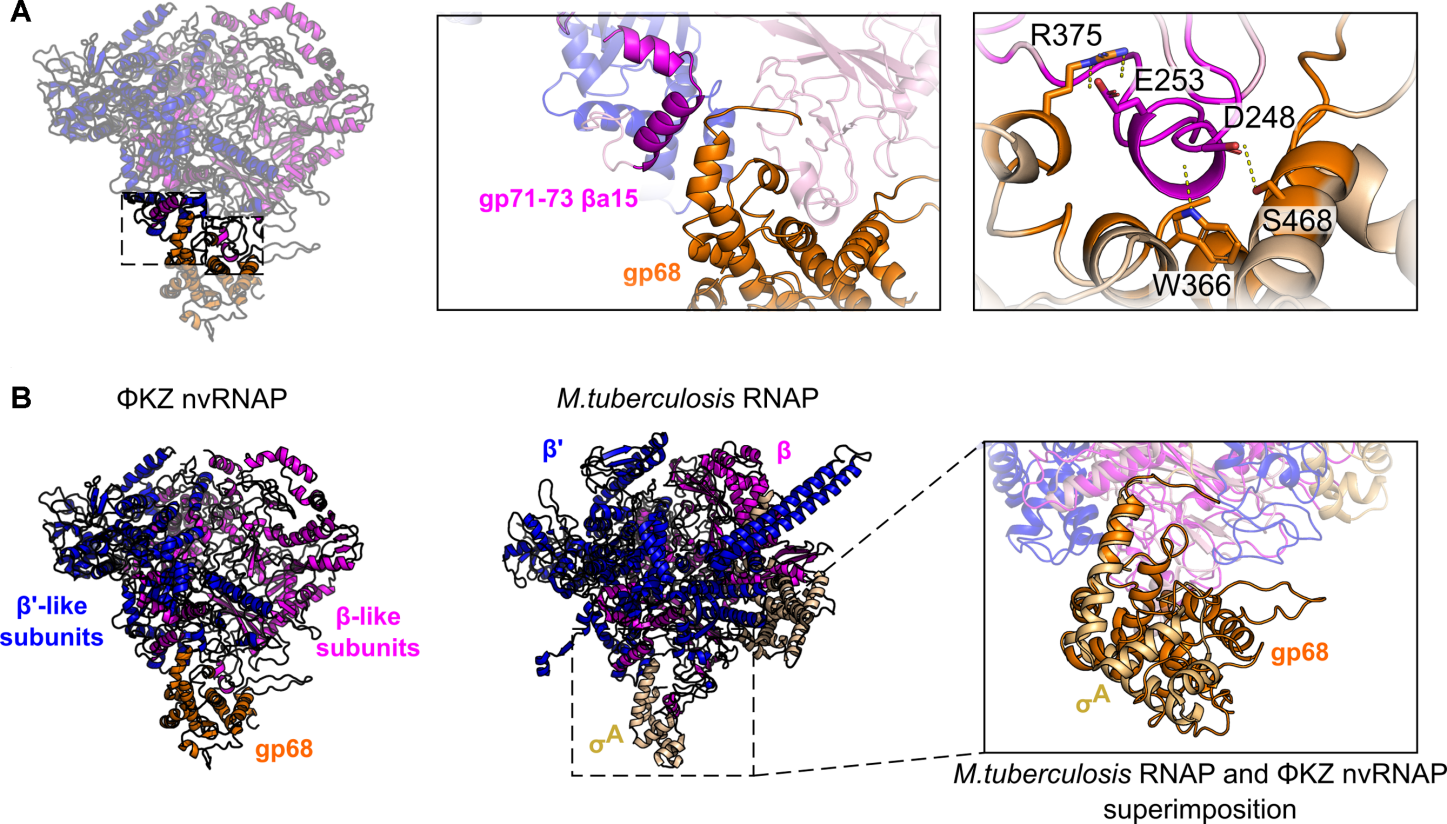
Gp68 is positioned identically to, and exhibits structural homology to, eubacterial σ-factor. (**A**) Views of the interaction surface and mode of gp68 with gp71–73 (cartoon representation with residues highlighted as sticks) (**B**) (left) Comparisons of *M. tuberculosis* (Mtb) RNAP (PDB 6EDT) and ΦKZ nvRNAP, showing the interaction surfaces between the two, and (right) superimpositions indicating colocation of gp68 and bacterial σ^A^ factor.

The five-subunit ΦKZ nvRNAP complex is sufficient to initiate transcription without α or ω accessory subunits (10,21). When we assembled full nvRNAP *in vitro* through the addition of independently purified gp68 to 4-subunit (4s) complexes (including only the four β/β’-like subunits) the resulting complexes contained all five nvRNAP components and successfully bound a long dsDNA containing the specific promoter consensus (Supplementary Table S4) in a similar manner to *in vivo* 5s assembled complexes (Figure 2A). They proved unable to initiate transcription from an RNA-DNA hybrid, however (Figure 2B). This implied that gp68 is included in the complex at the earliest stage of assembly. We also conducted subunit co-purification experiments in which we co-expressed pairs of subunits with one affinity-tag and assayed for recovery of both proteins. These showed that gp68 bound gp71–73 and gp55 (Supplementary Table S2), which was consistent with our structure in which the fifth subunit, gp68, is integrated into the nvRNAP making extensive contacts with gp71-73, and also contacting gp55 (Figure 1, Supplementary Table S3). The interaction between gp68 and both subunits is mostly governed by hydrogen bonds, with 4% and 14% of the solvent-accessible area of gp68 contributing to interactions with gp55 and gp71–73 respectively (Figure 3A). In eubacterial msRNAPs, the dimerised α subunits serve as an organizing platform for the assembly of a catalytically active core. Both β and β’ interact with the α-dimer, with universally conserved regions βa14 and βa15 being essential (33). In our structure, βa15 interacts with gp68 (Figure 3A), suggesting that gp68 at least contributes to the stabilisation of the catalytic core. This observation is consistent with the requirement for gp68 during the assembly process in order to form a catalytically active polymerase.

Superimposition of the nvRNAP structure with that of eubacterial RNAPs (using *M. tuberculosis* RNAP as an exemplar) revealed that the C-terminus of gp68 is located in the same position within the complex as the C-terminus of σ factor (Figure 3A). Its binding site on nvRNAP matches almost exactly that of region 4 of σ factor. Excess poorly ordered density that we assign to gp68 is also found to occupy other regions corresponding to eubacterial σ factor (Supplementary Figure S6). Furthermore, DALI analysis of the gp68 three-dimensional structure showed that the extended segment corresponding to region 4 has a weakly similar fold to eubacterial σ factors, although this structural homology lies within a more N-terminal region of σ factor, making its significance difficult to gauge (Supplementary Table S5A/B; Figure S7). While any of these observations made in independence would not necessarily be sufficient evidence to draw such a conclusion, from their observation in combination we surmise that gp68 has evolved from an ancestral σ factor, but has diverged to a great degree.

The gp68 C-terminus is substantially expanded in comparison to the C-terminus of eubacterial σ factor, making a substantially larger interaction (2308 versus 1495 Å^2^), and anchoring gp68 steadily within the complex. There is no consistent –35 sequence in the nvRNAP promoter, and therefore it is quite possible that this region of gp68 performs a mainly integrative and stabilising role, which is in keeping with the electrostatics of this region (Supplementary Figure S5), while the N-terminus would be intimately involved in DNA binding. Previously, we have shown that the absence of gp68 compromises the assembly of a full complex, as well as the binding of the enzyme to DNA-templates and transcription initiation (Figure 2) (21). To determine which subunits are involved in DNA binding, we carried out DNA binding assays with ΦKZ nvRNAP. We established binding by electrophoretic mobility shift assay, and then used UV-crosslinking to covalently link the bound DNA to the adjacent proteins. We treated the crosslinked samples with SDS to separate the polypeptides and crosslinked DNA within the complex. We then separated the resulting mixture by electrophoresis, allowing the identification of several DNA-linked breakdown intermediates. Mass spectrometry identified gp123 and gp68 within the band corresponding to the fully denatured complex containing the DNA binding subunits. This confirmed the involvement of gp68 in DNA binding (Figure 2C). Based upon this observed involvement of gp68 in DNA binding, we then carried out limited proteolysis with two different DNA templates, short DNA (from -12 to +15 nucleotide on non-template strand; Supplementary Table S4), and a long DNA (from –64 to +148 bp; Supplementary Table S4). In the absence of DNA, limited proteolysis released the N-terminus of gp68 (1–382). The presence of the short junction DNA led to a weak protective effect, while the presence of the longer dsDNA was strongly protective against gp68 proteolysis (Figure 2D and Supplementary Figure S8). The protection of gp68 from proteolysis by DNA confirms the crucial role of the fifth subunit in DNA binding, and is congruent with σ factor-like activity. Moreover, if the DNA incorporated was long enough its binding with nvRNAP resulted in a conformational change to a more compact complex.

Overall, our results imply that ΦKZ nvRNAP has evolved a divergent, minimal, and elegant structure to allow the assembly of a functional RNAP without the canonical α and ω subunits. It has also developed an embedded subunit involved intimately in both the assembly of the catalytically active enzyme and DNA recruitment, that most likely began as an ancestral σ-factor. The exchange of σ-factor for alternate promoter selection presumably became unnecessary for ΦKZ’s lifecycle. Given the substantially greater divergence of gp68 from eubacterial enzymes in comparison to the remaining members of the complex, we would surmise that the ancestor of gp68 was transferred before those of the β/β’-like subunits, acting as a viral σ-factor.

There are only a few exceptional bacteriophages known to encode multi-subunit RNAPs, including bacteriophages PBS2 (9,34) and AR9 (35). AR9 also encodes distant homologues to β and β’ subunits whose gene products assemble into two msRNAPs (35), and a fifth subunit, gp226, crucial for promoter recognition (11), suggesting a similar mechanism to ΦKZ nvRNAP. The AR9 complex remains catalytically active in the absence of the fifth subunit, however, which suggests a less intimate integration into the msRNAP complex than has been achieved by ΦKZ nvRNAP.

The conservation of the key elements of the eubacterial catalytic core in ΦKZ implies that processive polymerisation and catalysis will proceed similarly; ΦKZ has evolved an extremely streamlined msRNAP, which has no reliance on α or ω subunits, or indeed any additional stabilising contacts in their place within the complex, however the nuts and bolts of the processive polymerase are all retained as in their eubacterial orthologues. The assembly and stabilisation of the active site are likely to be divergent, however, as our biochemical results imply both to be gp68 dependent. Given the extreme divergence of gp68 from σ factor and its embedding within the complex, promoter recognition and bubble formation are also unlikely to proceed identically. Further studies of the ΦKZ nvRNAP will be required to understand how these key roles are achieved.

## DATA AVAILABILITY

The cryo-EM density maps resolved for the ΦKZ nvRNAP complex have been deposited in the EM Databank under accession codes EMD-12885 and EMD-12886, while the corresponding molecular models have been deposited in the protein data bank as PDB-IDs 7OGP and 7OGR. Electron micrographs allowing reproduction of our structural results have been deposited as EMPIAR-10707.

## Supplementary Material

gkab539_Supplemental_File
