# Correction to ‘Structure of the bacteriophage PhiKZ non-virion RNA polymerase’

**DOI:** 10.1093/nar/gkab846

**Published:** 2021-09-16

**Authors:** Natàlia de Martín Garrido, Mariia Orekhova, Yuen Ting Emilie Lai Wan Loong, Anna Litvinova, Kailash Ramlaul, Tatyana Artamonova, Alexei S Melnikov, Pavel Serdobintsev, Christopher H S Aylett, Maria Yakunina

**Affiliations:** Section for Structural and Synthetic Biology, Department of Infectious Disease, Imperial College London, London, UK; Peter the Great St. Petersburg Polytechnic University, St. Petersburg, Russia; Section for Structural and Synthetic Biology, Department of Infectious Disease, Imperial College London, London, UK; Peter the Great St. Petersburg Polytechnic University, St. Petersburg, Russia; Section for Structural and Synthetic Biology, Department of Infectious Disease, Imperial College London, London, UK; Peter the Great St. Petersburg Polytechnic University, St. Petersburg, Russia; Peter the Great St. Petersburg Polytechnic University, St. Petersburg, Russia; St. Petersburg State University, St. Petersburg, Russia; Section for Structural and Synthetic Biology, Department of Infectious Disease, Imperial College London, London, UK; Peter the Great St. Petersburg Polytechnic University, St. Petersburg, Russia; Sechenov Institute of Evolutionary Physiology and Biochemistry Russian Academy of Sciences, St. Petersburg, Russia

*Nucleic Acids Research*, 2021, 49(13): 7732–7739, https://doi.org/10.1093/nar/gkab539

In the originally published version of this manuscript, there was a spelling error in the first author's name. The name should read: Natàlia de Martín Garrido.

This error has been corrected online.

