# Supplementary material for: Structure of the bacteriophage PhiKZ non-virion RNA polymerase
Source: Nucleic Acids Res. 2021 Jun 28;49(13):7732–9. doi: 10.1093/nar/gkab539 (PMC8287921; doi:10.1093/nar/gkab539)
Supplement: gkab539_Supplemental_File [file gkab539_supplemental_file.pdf]

Supplementary Materials

Structure of the Bacteriophage PhiKZ non-Virion RNA Polymerase

Natàlia de Martín Garrido[1]*, Mariia Orekhova[2]*, Yuen Ting Emilie Lai Wan Loong[1], Anna Litvinova[2], Kailash Ramlaul[1], Tatyana Artamonova[2], Alexei S. Melnikov[2], Pavel Serdobintsev[3], Christopher H. S. Aylett[1]† & Maria Yakunina[2,4]†

[1] Section for Structural and Synthetic Biology, Department of Infectious Disease, Imperial College London, London, United Kingdom.

[2] Peter the Great St. Petersburg Polytechnic University, St. Petersburg, Russia

[3] St. Petersburg State University, St. Petersburg, Russia

[4] Sechenov Institute of Evolutionary Physiology and Biochemistry Russian Academy of Sciences, St. Petersburg, Russia

* These authors contributed equally to this study.

† To whom correspondence may be addressed:

CHSA c.aylett@imperial.ac.uk

MY yakuninam@nanobio.spbstu.ru

Supplementary Figures

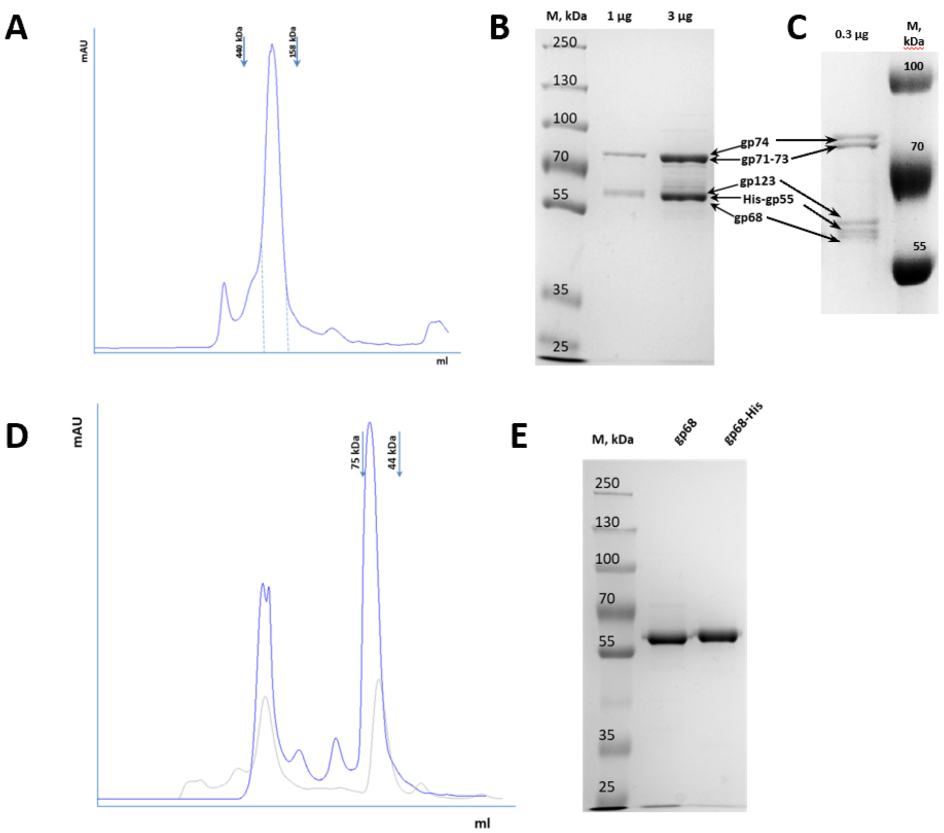

Supplementary Figure 1: Purification of recombinant ΦKZ nvRNAP and gp68. The size-exclusion column chromatogram (A) and SDS-PAGE analysis (B and C) of nvRNAP samples. The area of nvRNAP (nvP) on A is indicated by dotted-lines. On B and C arrows indicate the subunits of nvRNAP corresponding to mass-spectrometry results. The size-exclusion column chromatogram (D) and SDS-PAGE analysis (E) of gp68 and gp68-His samples. On D the dotted lines indicate gp68 (grey line) and gp68-His (blue line) monomers. For SDS-PAGE analysis 1 µg of each purified protein was used.

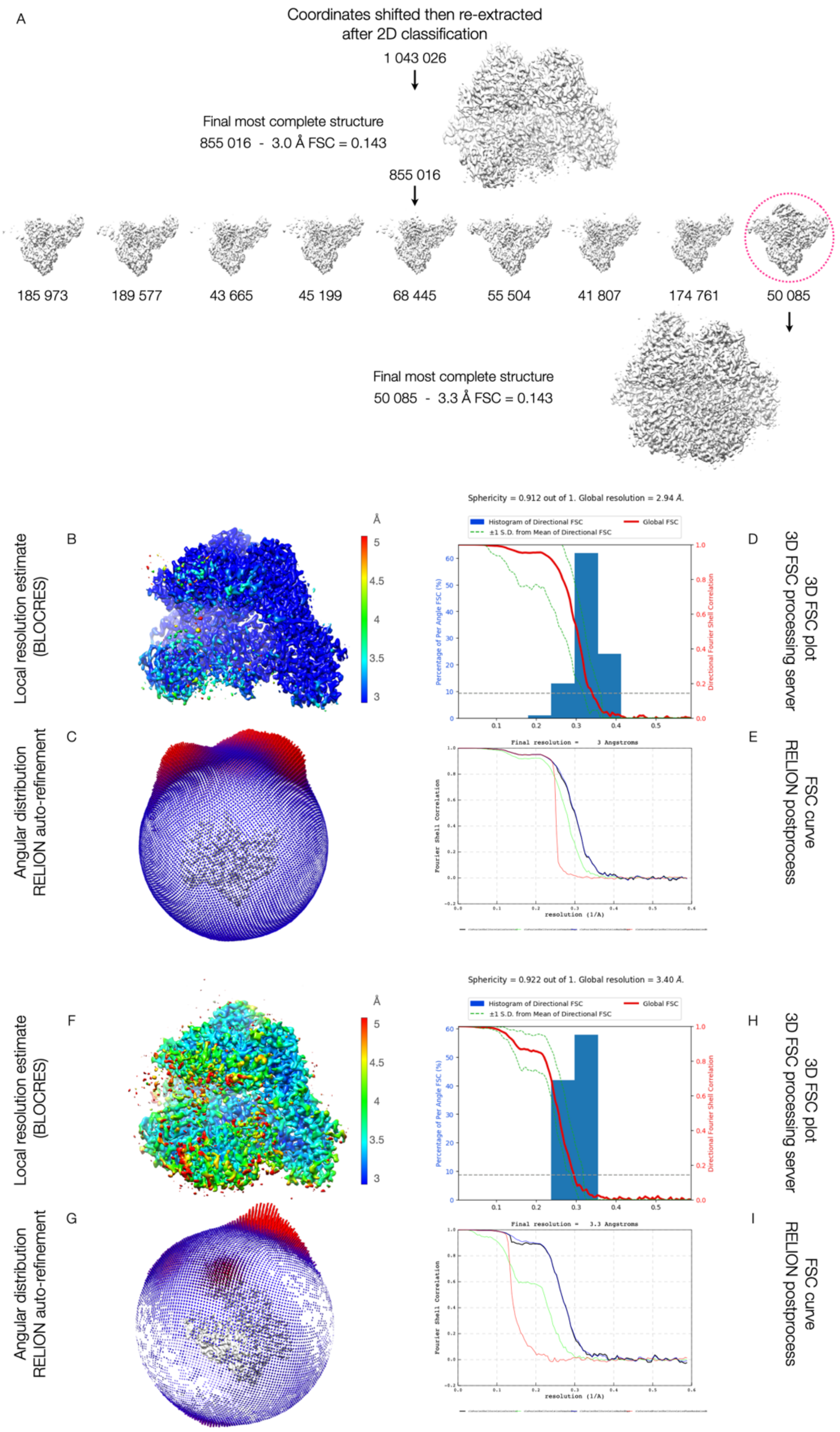

Supplementary Figure 2: Cryo-EM data processing scheme. A) Flow-chart depicting the data processing procedure. Particle numbers retained at each stage of the process are indicated below each 3D reconstruction. Local resolution map calculated using BLOCRES for core and clamp (B/F). Angular distribution plots of the core and clamp reconstructions (C/G). Core and clamp (D/H) 3D FSC analysis plot, 1 σ interval, and histogram. Global FSC curves for the final core and clamp maps (E/I).

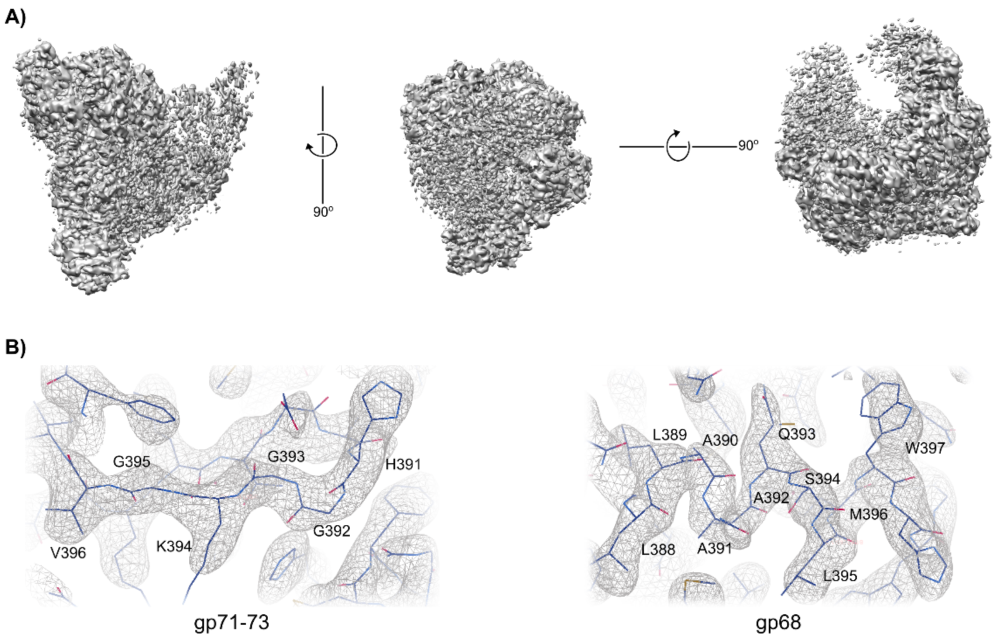

Supplementary Figure 3: High resolution features of nvRNAP cryo-EM map. Cryo-EM density of nvRNAP at 2.98 Å resolution. B) Molecular model fitted into high-resolution map. Molecular model is displayed in blue with side chains, and the map is displayed as a grey mesh.

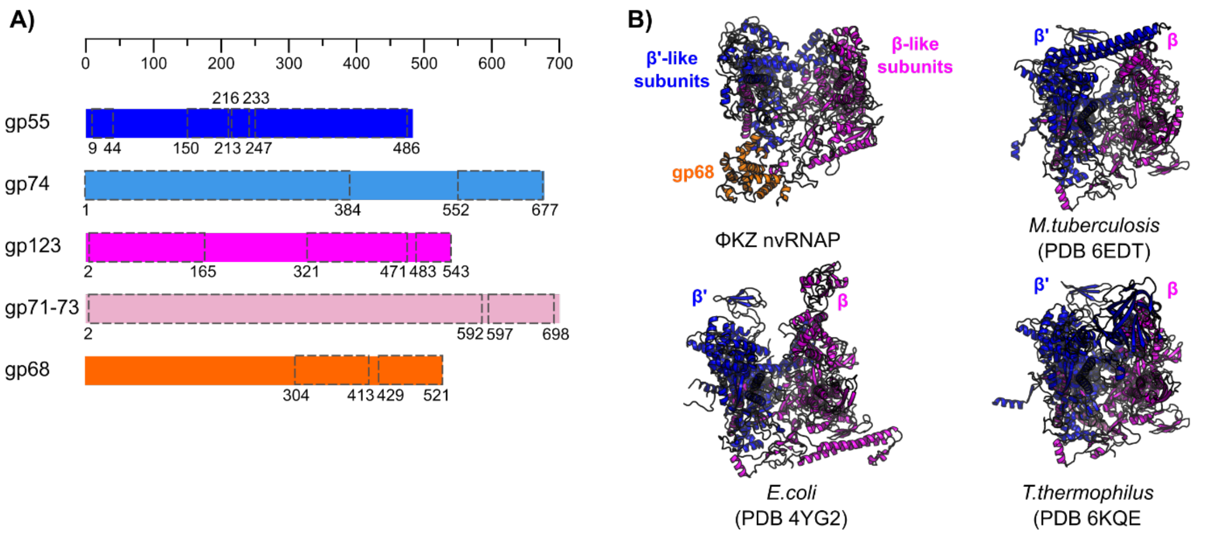

Supplementary Figure 4: Schematic diagram of the nvRNAP molecular model and structural comparison to eubacterial msRNAPs. A) Each box represents the sequence of each nvRNAP subunit, following the same colour scheme than in structural representations. Grey boxes with dotted line represent the regions for which a molecular model has been built. The numbers on top or below the boxes represent the boundaries for each built region. B) Structural comparison of nvRNAP and eubacterial RNAPs. Bacterial β subunits are depicted and blue while β' subunits are depicted in magenta. For nvRNAP β-like subunits are depicted in blue and β'-like subunits are depicted in magenta.

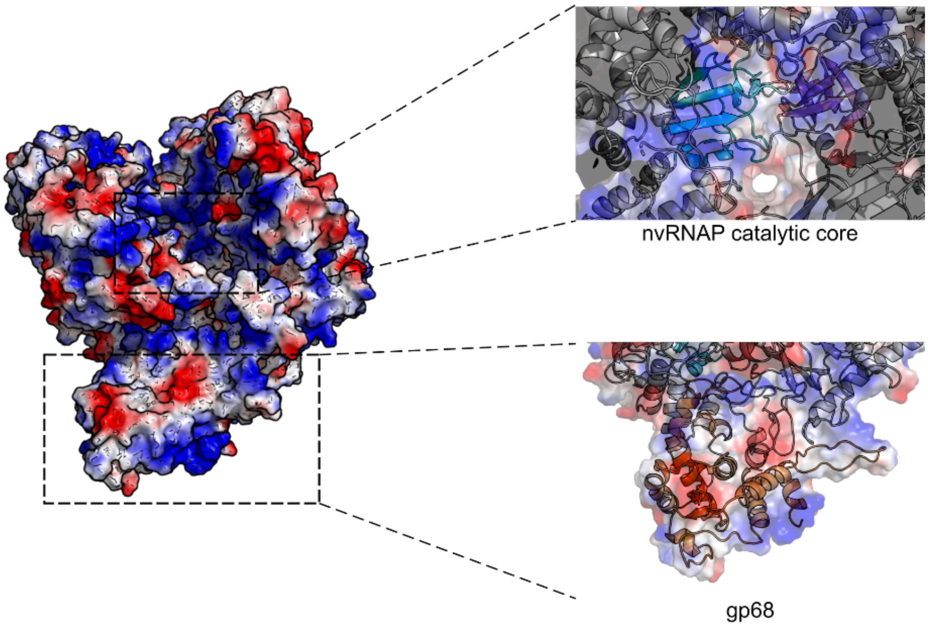

Supplementary Figure 5: Electrostatic potential surface of nvRNAP. On the left, electrostatic potential surface of the nvRNAP molecular model with blue regions representing positive charges, red representing negative and white neutral. The top inset displays the nvRNAP catalytic core with fitted cartoon and the bottom inset represents gp68 coloured in orange.

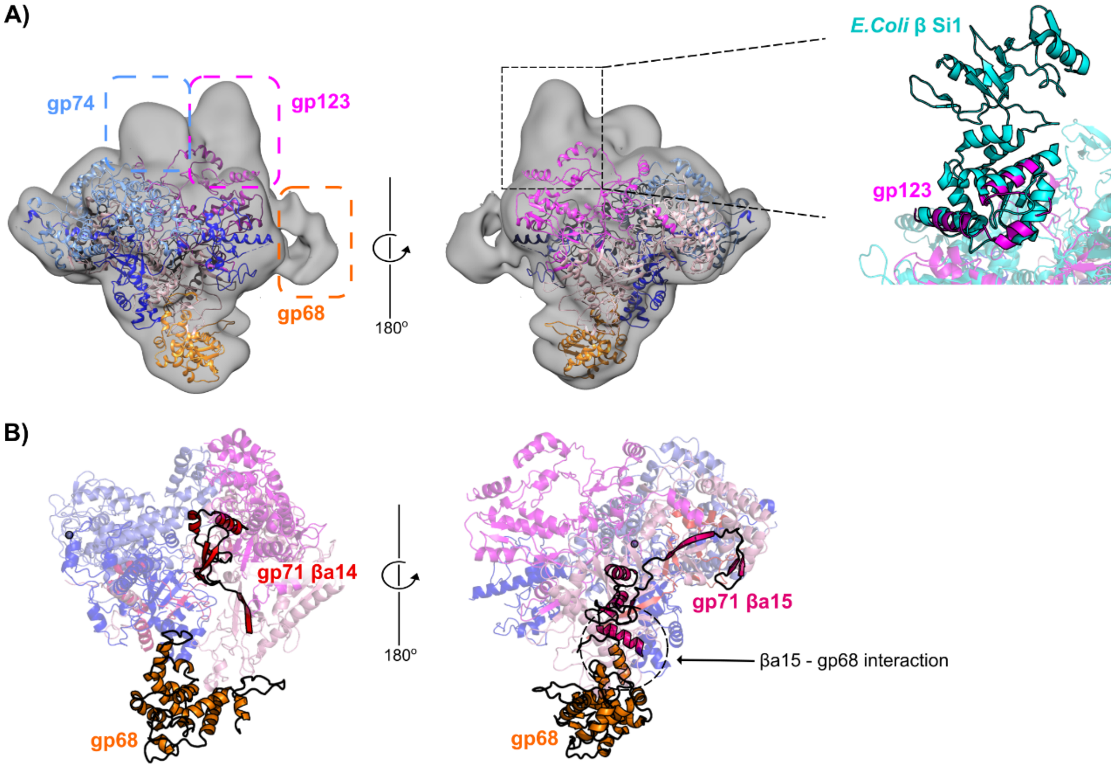

Supplementary Figure 6: Low resolution features of cryo-EM structure of nvRNAP and gp68 interaction. A) Three-dimensional map with a gaussian filtered applied coloured in light grey, with fitted nvRNAP structure represented in cartoon. Gp55 is depicted in blue, gp74 in light blue, gp71-73 in light pink, gp123 in magenta and gp68 in orange. Extra density is highlighted in dotted-line rectangles coloured accordingly with the subunit they are expected to correspond to. On the right, the inset shows superimposition of gp123 on Si1 region of *E. coli* RNAP. B) Cartoon representation of nvRNAP with gp71-73 βa14 and βa15 highlighted. Interaction of βa15 and gp68 is pointed with an arrow.

A)

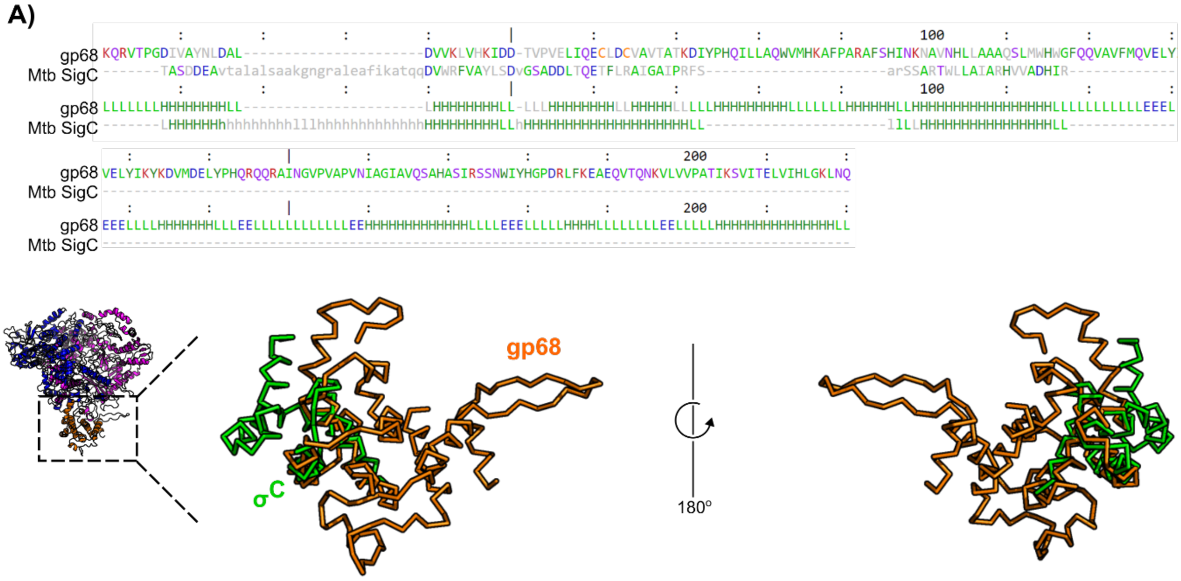

B)

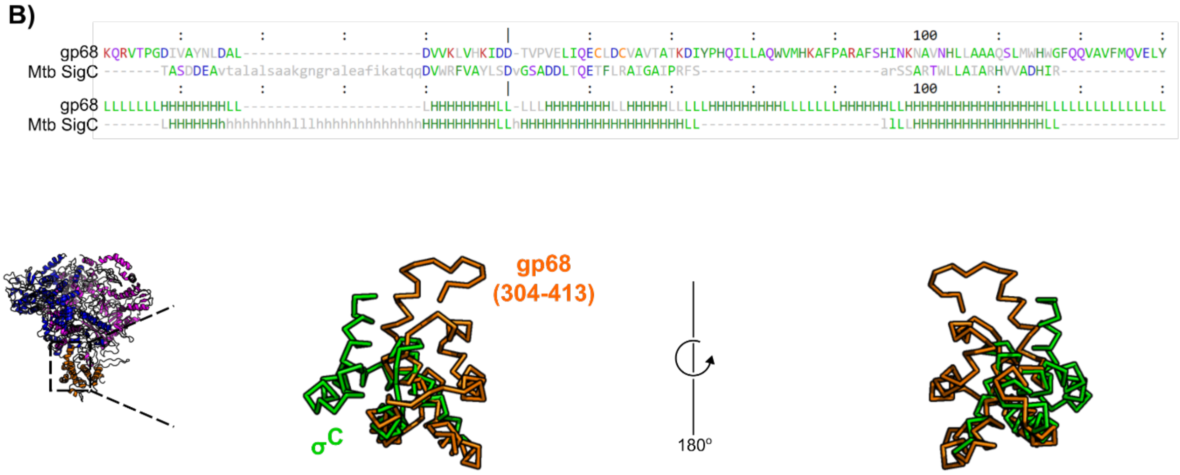

Supplementary Figure 7: Structural alignment of gp68 and *M. Tuberculosis* sigma factor C. Sequence alignment based on structural similarity made with DALI. The top part shows the amino acid sequences of gp68, Mtb σ^{c} and Mtb σ^{J}. Below, secondary assignments are represented with H (helix), E (strand) and L (coil). The most frequent amino acid type is coloured in each column. Superimposition of Mtb σ^{J} (PDB: 5XE7) on gp68 depicted in ribbon representation. Panel (A) shows the results for the full modelled sequence of gp68 and (B) shows the results for residues 304-413 of gp68.

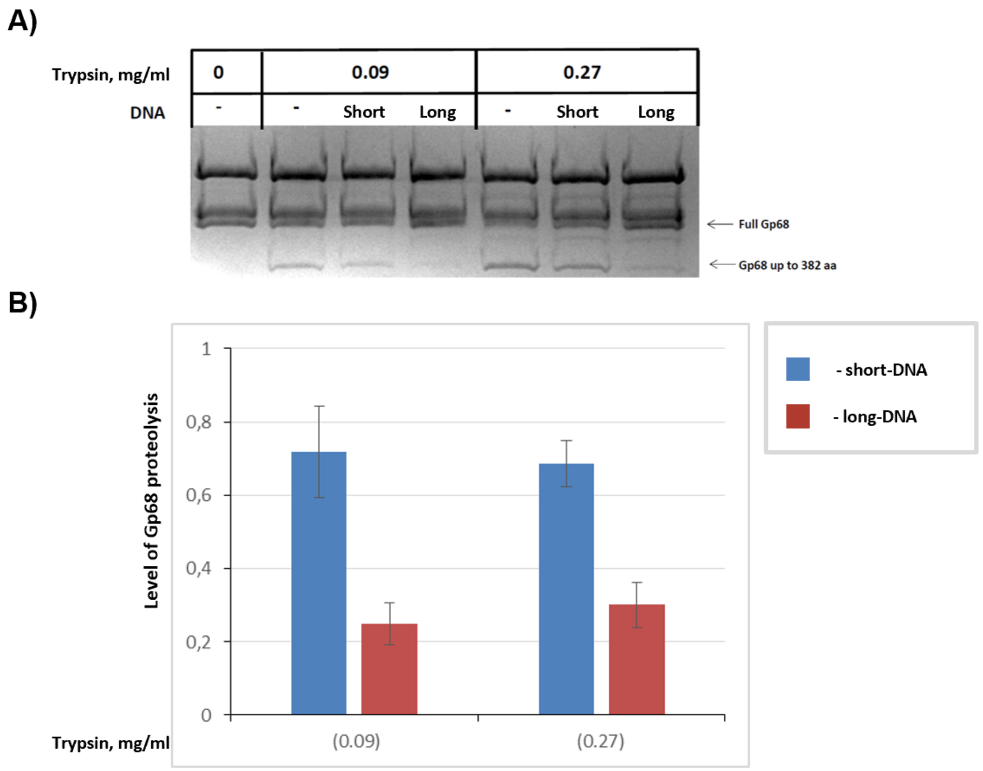

Supplementary Figure 8: Quantification of proteolytic protection by DNA. A) Trypsin limited proteolysis of 5s nvRNAP without and with two types of DNA templates, short and long (see Supplementary Table 4 for details). The full Gp68 and the fragment of Gp68 up to 382 aa are marked by arrows. B) Histogram indicating the levels of gp68 proteolysis by trypsin in nvRNAP complexes with short (blue) or long (red) DNA templates. The intensity of gp68 proteolysed fragment band in the control sample without DNA was taken as unity in each case. The level of gp68 proteolysis in DNA-nvRNAP complexes was calculated as the ratio of the band intensity of the proteolysed fragment in the complex with DNA to the band intensity of the proteolysed fragment in the nvRNAP without DNA. The data shown are the mean values from three independent measurements, and the error bars represent the standard deviations.

Supplementary Tables

	ΦKZ nvRNAP<br>EMD-12886	ΦKZ nvRNAP including clamp<br>EMD-12885
Magnification (×)	48,000	48,000
Voltage (kV)	300	300
Electron exposure (e^{-}/Å^2)	51	51
Defocus range (μm)	-0.75 to -3.25	-0.75 to -3.25
Pixel size (Å/pix)	0.85	0.85
Symmetry imposed	C1	C1
Initial number of particles	3 179 799	3 179 799
Final number of particles	855 016	50 085
Map resolution (Å) at FSC = 0.143	3.0	3.3
	**PDB ID 7OGR**	**PDB ID 7OGP**
Mean model-map correlation / B-factor	0.8032 / 40.261	0.8360 / 109.761
Molprobity overall score / clash score	1.5 / 3.80	1.44 / 3.33
RMS deviations in bond lengths / bond angles	0.003 Å / 0.720°	0.003 Å / 0.689°
Ramachandran favoured / allowed / disallowed (%)	95.31 / 4.69 / 0.00	95.37 / 4.63 / 0.00
Rotamers favoured / allowed / disallowed (%)	95.55 / 4.38 / 0.07	95.39 / 4.34 / 0.27

		N° of residues			
**Subunit**	**Chain ID**	**Model**	**Sequence**	**% Modelled Sequence**	**Modelled sections**
gp55	A	358	489	73	9-44, 150-213, 216-233, 247-486
gp74	D	510	677	75	1-384, 552-677
gp71-73	C	693	700	99	2-592, 597-698
gp123	E	370	543	68	1-165, 322-471, 483-543
gp68	B	203	521	39	304-413, 429-521
Total	-	2134	2930	73	

Supplementary Table 1: Structural model. Data collection, processing and model building statistics.

	gp55	gp68	gp71-73	gp74	gp123
**His-gp55**		++	+	-	-
**Gp68-His**	(++)		+++	-	(-)
**His-gp71-73**	(+)	(+++)		-	(-)
**His-gp74**	(-)	(-)	(-)		(-)
**His-gp123**	(-)	-	-	-	
**(His-gp55)-gp71-73**		+		-	+
**(gp68-His)-gp71-73**	+			-	+

Supplementary Table 2: *In vivo* Co-purification experiments. The result is represented in the cell at the intersection of the column and row. Interacting pairs are marked +, non-interacting -. In the case of pairs that have not been verified directly, the + or - symbols are bracketed and displayed according to the data obtained for a similar pair.

				Gp68 or σ interactions	
	Total solvent-accessible surface area (Å2)	Total buried-surface area (Å2)	% of buried-surface area	Interacting partner	Interface buried surface area (Å2)
**nvRNAP**	131,707.40	20,403.30	**15.49**	gp71-73	1,862.10
				gp55	445.90
				**Total**	**2,308.00**
**6EDT**	128,108.00	9,775.30	**7.63**	β	1,130.90
				β'	364.50
				**Total**	**1,495.40**

Supplementary Table 3: Comparison of buried surface area of nvRNAP gp68 and σ factor A from *M. tuberculosis*. Values extracted from PDBePISA server (https://www.ebi.ac.uk/pdbe/pisa/)

<table>
<tr><td>The DNA-template type</td><td>Oligonucleotide name</td><td>Sequence</td></tr>
<tr><td rowspan="3">DNA-RNA hybrid</td><td>Cy3-RNA</td><td>5’-Cy3-GUAGCGGA-3’</td></tr>
<tr><td>Template DNA</td><td>5’-GGTCCTGTCTGAAATTGTTATCCGCTAC-3’</td></tr>
<tr><td>Non-template DNA</td><td>5’-ACAATTTCAGACAGGACC-3’</td></tr>
<tr><td colspan="3">5’-ACAATTTCAGACAGGACC-3’
5’-GUAGCGGA-3’
3’-CATCGCCTATTAAAGTCTGTCCTGG-5’</td></tr>
<tr><td rowspan="2">Long DNA (from -64 to +148)</td><td>KZP119F</td><td>5’-GAGATGTACAGTGTATCATTTAGATAGC-3’</td></tr>
<tr><td>KZ119prex</td><td>5’-TCGGAAACTATATAGTTGGTGTCTAAC-3’</td></tr>
<tr><td colspan="3">+1
5’GAGATGTACAGTGTATCATTTAGATAGCTAGTAATTTTAGTGAATGTATTTGCTATATTGCTATGTAGACAGTTCCCAAAAGCC
TAAAGTTACAATATAGGTACTTTCACAATGACCCAAGTTCTGGTTATGCCAAAAGCTGTTTTTGAATCATATAAAACAGCAGGTGT
ACAATTTAACAAATTGTTAGACACCAACTATATAGTTTCCGA 3’</td></tr>
<tr><td rowspan="2">Short DNA (from -12 to +15 on template strand)</td><td>KZP119_12t</td><td>5’-TTGGGAACTGTCTACATAGCAATATAG-3’</td></tr>
<tr><td>FJ.KZP119(+3)nt</td><td>5’-AGACAGTTCCCAA-3’</td></tr>
<tr><td colspan="3">+1
5’-AGACAGTTCCCAA-3’
3’-GATATAACGATACATCTGTCAAGGGTT-5’</td></tr>
</table>

Supplementary Table 4. List of the nucleotides used for DNA-templates preparation. For each template, the list of oligonucleotides used for preparation and the full sequence are given. The RNA-oligo is italic in the DNA-RNA hybrid sequence. In the phiKZ nvRNAP specific templates, the promoter consensus sequences are marked by red colour. The last nucleotide in the 5'-TATG-3' consensus sequence is the transcriptional start point (+1).

PDB ID	Z-score	RMSD	% Identity	Description
6cgh-A	4.1	3.1	4	DNAJ HOMOLOG SUBFAMILY C MEMBER 2;
1k30-A	3.9	2.7	7	GLYCEROL-3-PHOSPHATE ACYLTRANSFERASE;
4noo-B	3.9	3.9	4	VGRG PROTEIN;
6z8k-A	3.8	4.3	4	LA CROSSE VIRUS 5' VRNA 1-10;
5dbk-A	3.8	4	9	TRANSCRIPTIONAL REGULATOR/TPR DOMAIN PROTEIN;
3jck-F	3.7	3.7	5	26S PROTEASOME REGULATORY SUBUNIT RPN3;
4xxk-A	3.7	10.3	11	PHYCOBILIPROTEIN APCE;
3bu8-B	3.7	4.7	4	TELOMERIC REPEAT-BINDING FACTOR 2;
6mit-G	3.7	13.8	8	LIPOPOLYSACCHARIDE EXPORT SYSTEM ATP-BINDING PROT
6ans-B	3.7	5	0	UNCHARACTERIZED PROTEIN;
3urz-B	3.7	3.5	5	UNCHARACTERIZED PROTEIN;
4zlh-B	3.7	3.5	4	LIPOPOLYSACCHARIDE ASSEMBLY PROTEIN B;
5hzd-A	3.6	3.9	6	3' TERMINAL URIDYLYL TRANSFERASE;
**2o7g-A**	**3.6**	**3**	**17**	**PROBABLE RNA POLYMERASE SIGMA-C FACTOR;**
6ac0-A	3.6	9	2	TUMOR NECROSIS FACTOR RECEPTOR TYPE 1-ASSOCIATED
5cwc-A	3.6	2.4	9	DESIGNED HELICAL REPEAT PROTEIN;
2hh6-A	3.6	2.3	8	BH3980 PROTEIN;
1gzs-B	3.5	11	14	SOPE;
4yvo-A	3.5	3.8	8	PROTEIN FLUORESCENT IN BLUE LIGHT, CHLOROPLASTIC;
6pir-B	3.5	3.9	2	MAVE;

Supplementary Table 5A: DALI server results for gp68. DALI server results using the full modelled sequence of gp68 showing. The table shows the top 20 molecules with highest Z-score.

PDB ID	Z-score	RMSD	% Identity	Description
**2o7q-A**	**4.1**	**3**	**17**	**PROBABLE RNA POLYMERASE SIGMA-C FACTOR;**
5jwy-A	3.9	3.1	8	PHOSPHATIDYLGLYCEROPHOSPHATASE B;
7c0q-A	3.9	3.1	5	EFFECTOR LPG2505;
6n8a-B	3.9	3.6	3	TRANSCRIPTION REGULATOR ACAB;
3n71-A	3.9	3.9	8	HISTONE LYSINE METHYLTRANSFERASE SMYD1;
1hz4-A	3.8	3.2	7	MALT REGULATORY PROTEIN;
2nn4-A	3.8	2.4	4	HYPOTHETICAL PROTEIN YQGQ;
6jbq-F	3.7	3.5	10	DNA-DIRECTED RNA POLYMERASE SUBUNIT ALPHA;
6n8e-A	3.7	6.7	9	HOLO-OBIF1;
4elj-A	3.7	5.4	4	RETINOBLASTOMA-ASSOCIATED PROTEIN;
5ydn-A	3.6	3.5	5	GENE PRODUCT J;
6pss-L	3.6	4.6	10	DNA-DIRECTED RNA POLYMERASE SUBUNIT ALPHA;
1qte-A	3.6	3.5	6	SOLUBLE LYTIC TRANSGLYCOSYLASE SLT70;
5dbk-A	3.6	3.3	15	TRANSCRIPTIONAL REGULATOR/TPR DOMAIN PROTEIN;
1e7d-A	3.6	4.5	4	RECOMBINATION ENDONUCLEASE VII;
2pv4-A	3.5	3.8	14	UNCHARACTERIZED PROTEIN;
1l1l-A	3.5	3.2	2	RIBONUCLEOSIDE TRIPHOSPHATE REDUCTASE;
5t76-A	3.5	4	3	ALANINE--TRNA LIGASE, CYTOPLASMIC;
2qfc-A	3.5	3.3	6	PLCR PROTEIN;
3kdw-A	3.5	7.2	14	PUTATIVE SUGAR BINDING PROTEIN;

Supplementary Table 5B: DALI results for gp68. DALI server results using the N-terminus fragment of gp68 (304-413). The table shows the top 20 molecules with highest Z-score.
